# Control of parenteral blood stream infections in patients who need parenteral nutrition

**DOI:** 10.2478/abm-2024-0014

**Published:** 2024-06-28

**Authors:** Editorial Office of Asian Biomedicine

**Affiliations:** Faculty of Medicine, Chulalongkorn University, Bangkok 10330, Thailand

In many acute or chronic clinical conditions, parenteral nutrition (PN) is important for providing essential nutrients and calories to patients when enteral nutrition is inadequate or not acceptable [1]. However, PN has been associated with increased risks of thromboembolic, infectious, and metabolic complications [1, 2]. A multidisciplinary team has been advocated to administer PN partly due to the assumption that a high level of expertise is required for patient management, including the assessment of the patients' nutritional status and requirements [3]. Adequate monitoring is required to control metabolic complications such as inappropriate glycemic control, as well as the avoidance of electrolyte imbalances [4]. In addition, infectious complications also need close monitoring [4].

Some chronic conditions need long-term PN therapy. A recently drafted official guideline for providers and healthcare administrators has been available for providing safe home PN [5]. The guideline has addressed the indications for long-term PN therapy, central venous catheter (CVC) and infusion pump handling, infusion line and CVC site care, nutritional formulation and composition, and program monitoring and management [5].

Nutritional formulations have been improved over the years. Special formulations should be tailored for specific disease states (such as acute pancreatitis, surgery patients, critically Ill patients, inflammatory bowel disease) vascular access, and potentially expected complications of both short-term and long-term PN. PN is a mixture of solutions containing dextrose, amino acids, electrolytes, vitamins, minerals, and trace elements [4]. The exact composition and infusion rate are adjusted according to the nutritional and fluid needs of each patient.

Encountering catheter-related infection (CRI) is higher for patients requiring PN in hospital settings [6]. A systematic review and meta-analysis with trial sequential analysis revealed that although PN is not associated with greater mortality in hospitalized patients, it is associated with higher infectious complications [6]. Intravenous catheter-related blood stream infection (CRBSI) is a serious and potentially fatal complication of PN use [7]. This suggests that a strategy to deal with CRBSI in hospital settings is needed.

In this issue, Huang et al. [8] reported that the 28-day survival rates of *Candida* spp.-related CRBSI patients with timely catheter removal and/or appropriate antifungal treatment ranged from 45.45% to 88.89%. They suggested that patients with long-term catheter indwelling should be closely monitored, blood samples should be obtained immediately for bacterial or fungal culture upon the suspicious presentations of CRBSI, and CVC should be timely removed. For patients who still have had persistent mild-to-moderate inflammatory responses despite receiving high-grade sensitive antibiotics, antifungal treatment should be performed at the earliest event even when positive results are still pending.

Catheter impregnation, coating, or bonding of CVC have been proposed as another method to reduce CRI for patients who need PN treatment [9]. More research is needed to find a standard method for reducing CRI in patients obtaining PN.
